# Factors associated with inadequate treatment of syphilis during pregnancy: an integrative review

**DOI:** 10.1590/0034-7167-2021-0965

**Published:** 2022-09-09

**Authors:** Paula Marília Afonso Torres, Amanda Ribeiro de Paula Reis, Andressa Silva Torres dos Santos, Nádia Bruna da Silva Negrinho, Mayra Gonçalves Menegueti, Elucir Gir

**Affiliations:** I Universidade de São Paulo. Ribeirão Preto, São Paulo, Brazil

**Keywords:** Syphilis, Pregnancy, Therapeutics, Prenatal Care, Review, Sífilis, Embarazo, Terapéutica, Atención Prenatal, Revisión, Sífilis, Gravidez, Terapêutica, Cuidado Pré-Natal, Revisão

## Abstract

**Objectives:**

to analyze the evidence available in literature on factors associated with inadequate treatment of syphilis in pregnant women.

**Methods:**

an integrative review, carried out in the LILACS, CINAHL, Web of Science, Scopus, PubMed and EMBASE databases, with controlled descriptors therapeutic and prenatal syphilis.

**Results:**

nine publications composed the interpretative analysis, in which low education, income and maternal age, temporary lack of medication and HIV infection were associated with inadequate treatment of syphilis during pregnancy, in addition to delay or absence of prenatal care and receiving the 1^st^ dose of penicillin, lack of tests or treatment less than 30 days before childbirth, and partners’ low compliance with treatment.

**Final Considerations:**

among the main factors associated with inadequate treatment, clinical and sociodemographic aspects stand out, as well as failures in drug dispensing, prescription and monitoring of treatment of pregnant women and their partners by the health system.

## RESUMO

**Objetivos:**

analisar as evidências disponíveis na literatura sobre os fatores associados ao tratamento inadequado da sífilis em gestantes.

**Métodos:**

revisão integrativa, realizada nas bases de dados LILACS, CINAHL, *Web of Science*, Scopus, PubMed e EMBASE, com os descritores controlados sífilis gestantes terapêutica e pré-natal.

**Resultados:**

nove publicações compuseram a análise interpretativa, nas quais baixa escolaridade, renda e idade materna, falta temporária do medicamento e infecção por HIV foram associadas ao tratamento inadequado da sífilis na gestação, além do atraso ou ausência do pré-natal e no recebimento da 1ª dose de penicilina, falta de exames ou tratamento com menos de 30 dias antes do parto, e a baixa adesão do parceiro ao tratamento.

**Considerações Finais:**

dentre os principais fatores associados ao tratamento inadequado, destacam-se os aspectos clínicos da gestante, sociodemográficos, além de falhas na dispensação do medicamento, prescrição e acompanhamento do tratamento da gestante e do parceiro pelo sistema de saúde.

## RESUMEN

**Objetivos:**

analizar la evidencia disponible en la literatura sobre factores asociados al tratamiento inadecuado de la sífilis en gestantes.

**Métodos:**

revisión integradora, realizada en las bases de datos LILACS, CINAHL, *Web of Science*, Scopus, PubMed y EMBASE, con los descriptores controlados sífilis terapéutica embarazada y prenatal.

**Resultados:**

nueve publicaciones componían el análisis interpretativo, en el que la baja escolaridad, renta y edad materna, la falta temporal de medicación y la infección por VIH se asociaron con el tratamiento inadecuado de la sífilis durante el embarazo, además de la demora o ausencia de control prenatal y de recibir la 1^a^ dosis de penicilina, falta de exámenes o tratamiento menos de 30 días antes del parto, y baja adherencia al tratamiento por parte de la pareja.

**Consideraciones Finales:**

entre los principales factores asociados al tratamiento inadecuado se destacan los aspectos clínicos y sociodemográficos de la gestante, así como las fallas en la dispensación, prescripción y seguimiento del tratamiento de la gestante y su pareja por parte del sistema de salud.

## INTRODUCTION

Syphilis is a millennial sexually transmitted infection (STIs) caused by the bacterium *Treponema pallidum,* transmitted sexually and vertically during pregnancy or childbirth, when the treatment regimen of the diagnosed mother occurs inappropriately or does not occur. Vertical transmission of syphilis depends on the stages of maternal infection, the risk of which is higher during the primary and secondary stages of infection, being 70% to 100% in pregnant women who do not receive treatment and/or are treated inappropriately, with reduction in the latent and late phases (30%)^(1-2)^.

Among the main outcomes of gestational syphilis, evidenced in literature, there is an increased risk of fetal death by up to 21%, neonatal death, prematurity, underweight or congenital malformations^(3-4)^. It is worth mentioning that all adverse events of syphilis during pregnancy can be avoided with appropriate treatment during prenatal care, which consists in Brazil of penicillin G benzayene administration in a dose appropriate to the clinical phase diagnosed and started up to 30 days before childbirth, in addition to monthly follow-up to verify decreased titration. Pregnant women who do not meet these criteria are considered inadequately treated^(5)^.

Although it is a disease with affordable, effective and effective treatment, it still exhibits high incidence rates, representing a challenge for public health. The estimated worldwide prevalence of maternal syphilis in 2016 was 0.69%, 988,000 cases, with an overall congenital syphilis rate of 473 per 100,000 live births and 661,000 total cases^(6)^. In Brazil, in 2019, there were 61,127 cases of syphilis in pregnant women, a detection rate of 20.8 per 1,000 live births, and 24,130 cases of congenital syphilis, an incidence rate of 8.2 per 1,000 live births^(7)^.

Thus, in May 2016, the World Health Organization (WHO), through the World Health Assembly, adopted the global strategy 2016-2021, which defined priority actions to achieve goals for eliminating STIs by 2030, including congenital syphilis, and the expansion of evidence-based interventions and services to control STIs and reduce their impact as a public health concern^(7-8)^.

Some countries have already been certified by who as free of vertical transmission of syphilis. Among them, Cuba was the first country in the world to receive validation in 2015, later, in 2016 and 2017, about six Caribbean countries and territories such as Anguilla, Antigua and Barbuda, Bermuda, Cayman Islands, Montserrat and Saint Kitts and Nevis, plus the Americas, Thailand, Republic of Moldova and Belarus in 2016 and Malaysia in 2018^(9)^.

In Brazil, based on the criteria established by the Pan American Health Organization (PAHO) and WHO, adapted to the Brazilian reality, Boa Vista da Aparecida, municipality of Paraná State, achieved the Mother-to-child Congenital Syphilis Transmission Elimination Certificate. To this end, the municipality has reached the impact indicators in the last three years (incidence rate of syphilis ≤ 2.5 /1,000 live births in children under one year and less than 25% of children under one year with congenital syphilis) and process in the last 2 years (90% of pregnant women with four or more prenatal consultations, 90% of pregnant women diagnosed with syphilis who received a dose or more of penicillin and 50% or more of pregnant women diagnosed in the first trimester of pregnancy), in addition to assisting the others stipulated criteria^(10)^.

As a strategy to combat congenital syphilis, in 2021, Brazil launched the National Campaign to Combat Acquired and Congenital Syphilis, with the warning about the importance of prevention and early treatment, including as a target audience for pregnant women and their partners. As combat actions, the Mother-to-Child HIV and/or Syphilis Transmission Elimination Guide was launched, with the objective of standardizing the certification procedure in municipalities with 100 thousand or more inhabitants and in states, and a course on Comprehensive Care to People with STIs was conducted, with the purpose of offering professional qualification online^(11)^.

Therefore, given the data presented, including the high incidences of gestational syphilis, it is of paramount importance to recognize the factors associated with the occurrence of inadequate treatment, since it may direct public policies to certain risk groups. Thus, it is urgent to add a synthesis on the subject in question in a single study, in order to direct policies to improve prenatal care for pregnant women and their partners, thus reducing the number of cases of syphilis during pregnancy, with a consequent reduction in congenital syphilis and complications related to newborns.

## OBJECTIVES

To analyze the evidence available in literature on factors associated with inadequate treatment of syphilis in pregnant women.

## METHODS

### Study design

This is an integrative literature review, developed according to the following steps: selection of a question for review; sampling (search for studies according to inclusion and exclusion criteria); extraction of characteristics from primary research (data extraction); data analysis; interpretation of results; review report^(12)^. The Preferred Reporting Items for Systematic Reviews and Meta-Analyses (PRISMA) recommendations^(13)^ were followed.

### Data collection and organization

To elaborate the research question, the PICo strategy (P- Population) was used; I- Interest; Co- Context) was used. Subsequently, the Descriptors in Health Sciences (DeCS/BIREME) and the Medical Subject Headings (MeSH terms) were consulted, according to Chart 1. Thus, the following research question was constructed: what are the factors associated with inadequate treatment of syphilis in pregnant women?

**Chart 1:** PICo Strategy, DeCS and MESH terms

PICo Strategy			DeCS	MESH terms
PICo	Variables	Components		
**P**	**Population**	Pregnant women with syphilis	*Sífilis*	Syphilis
			*Gestantes* *Gravidez*	Pregnant Women Pregnancy
**I**	**Interest**	Inadequate treatment	*Terapêutica*	Therapeutics
**Co**	**Context**	Prenatal care	*Cuidado Pré-Natal*	Prenatal Care

The search for articles that made up this review took place in July 2021 in six databases, such as Latin American and Caribbean Literature in Health Sciences (LILACS), Cumulative Index to Nursing and Allied Health Literature (CINAHL), Web of Science, Sci Verse Scopus (Scopus), PubMed and EMBASE. For this, searches were performed respecting the singularities of each database, using the combination of Boolean operator “AND” between descriptors and Boolean operator “OR” between synonymous words. The search strategy employed for all databases was [(“Syphilis”) AND (“pregnant women” OR “pregnancy”) AND (“therapeutics”) AND (“Prenatal Care”)].

Articles available in full with research results that answered the question of the study and in all languages were included. Secondary studies (literature reviews, experience reports, reflection articles, editorials and letters), duplicate publications (duplicate manuscripts were considered only once) and productions not related to the purpose of this study were excluded. For the selection of articles, there was no time frame.

The results found in the searches were entered into the Rayyan web application, developed by Qatar Computing Research Institute (QCRI)^(14)^, to assist in article organization and selection. The reading of articles’ titles and abstracts and their selection were performed by two independent researchers. Subsequently, the selected articles were read in full in the first stage, and the relevant information was extracted with the help of an adapted instrument^(15)^ containing the following information: title; year of publication; objective; method (study design and site, participants, data collection and data analysis); main results of each article; and conclusion. It is worth noting that the disagreements between the selection of articles were resolved through agreement among researchers in the two stages.

The articles’ level of evidence was ordered through assessment of its methodological design, using the classification of seven levels: level I - evidence from a systematic review or meta-analysis of multiple randomized controlled clinical trials; level II - evidence from at least one well-designed randomized controlled clinical trial; level III - evidence derived from well-designed clinical trials without randomization; level IV - evidence derived from well-designed cohort and case-control research; level V - evidence from a systematic review using descriptive and qualitative methodologies; level VI - evidence from only one descriptive or qualitative study; level VII - evidence originating from authority concepts and/or expert committees report^(16)^.

### Data analysis

For data analysis, an analytical framework was constructed that allowed gathering and synthesizing the main information of included articles, as presented later. Data were interpreted and compared and later synthesized descriptively.

## RESULTS

**Figure 1:**
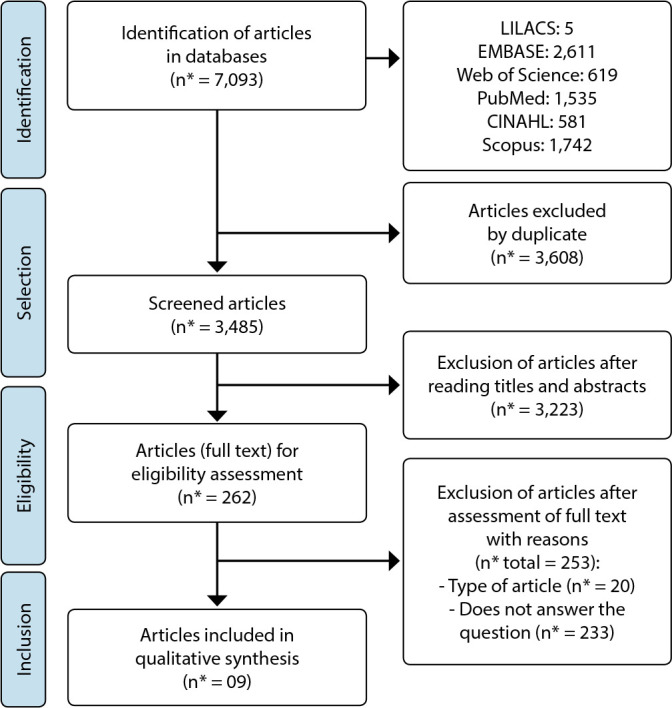
Search diagram and selection of articles according to PRISMA n* = number of articles.

The selection of articles found through the different word crosses followed PRISMA recommendations^(13)^, as shown in Figure 1.

The final sample consisted of nine articles that assessed the factors associated with inadequate treatment of syphilis in pregnant women. Most, seven, (77.7%) were published in international scientific journals, and only two (22.2%), in Brazilian journals. There was a predominance of eight (88.8%) studies with a quantitative approach, while only one (11.1%) was qualitative, and 66.6% had level VI evidence and were in English.

Still, regarding the study site, the largest portion, seven (77.7%), was developed outside Brazil, namely: Argentina, United States, Thailand, two in China and two in South Africa. In Brazil, two (22.2%) were carried out in the Northeast, in the states of Pernambuco and in Rio Grande do Norte.

The characteristics of articles included in this review, regarding the authors, journal, objectives, method and level of evidence, are described in Chart 2. The main results regarding the definition of adequate and inadequate treatment and factors associated with inadequate treatment, as well as the prevalence or incidence found and the profile of participants, are shown in Chart 3.

**Chart 2:** Characterization of articles selected for analysis according to authors/year, journal, objective(s), method and level of evidence

Authors/year	Journal	Objective (s)	Method (design, site, participants)	Level of evidence
Rotchfor K, et al, 2000^(17)^	Tropical Medicine International Health	Demonstrate the impact on perinatal mortality of inadequate treatment for maternal syphilis despite adequate screening.	**Design:** randomized controlled trial. **Site:** 12 clinics offering prenatal care in Hlabisa, KwaZulu, South Africa. **Participants:** 1,783 pregnant women tested for syphilis at their first prenatal visit.	II
Mullick S, 2005^(18)^	Sexually Transmitted Infections	Establish the degree of compliance with syphilis treatment.	**Design:** retrospective study. **Site:** Prince Mshiyeni Memorial Hospital, Umlazi Municipality, south of Durban, KwaZulu Natal. **Participants:** 18,128 prenatal records of women receiving prenatal care in the scenario in question.	VI
Brito ESV, et al, 2009^(19)^	*Revista de Atenção Primaria à Saúde*	Assess the quality of prenatal care in the municipality of Olinda using congenital syphilis as an indicator.	**Design:** descriptive, quantitative and cross-sectional study. **Site:** Olinda, in the Metropolitan Region of Recife, Pernambuco. **Participants:** epidemiological notification and investigation forms for cases of congenital syphilis, available in the Brazilian National Notifiable Diseases System (*Sistema Nacional de Agravos de Notificação*), 46 nurses and two epidemiological surveillance technicians.	VI
Zhu L, et al, 2010^(20)^	International Journal of Infectious Diseases	Assess trends and determinants of maternal and congenital syphilis in Shanghai, China.	**Design:** prospective cohort study **Site:** hospitals and clinics in Shanghai, China. **Participants:** 535,537 pregnant women who had their prenatal services and had their babies in Shanghai.	IV
Chávarro MAS, et al, 2017^(21)^	*Revista Mexicana de Pediatria*	Describe the factors related to failure in diagnosis and maternal treatment.	**Design:** case-control study conducted through a review of clinical records. **Site:** *Hospital Materno Infantil Maria Eva Duarte de Peron,* in Malvinas Argentina. **Participants:** postpartum women and live newborns at the research site, during the years 2014 and 2015. It was divided into two groups: **Cases**: live newborns of women diagnosed with untreated or inadequately treated syphilis who met the definition of congenital syphilis of the Ministry of Health of the Nation (Argentina). **Control**: live newborns of women without a diagnosis of syphilis.	IV
Hong F, et al, 2017^(22)^	IDSA - Infectious Diseases Society of American	Report the risk of developing congenital syphilis among infants born to mothers with different maternal syphilis treatment scenarios during pregnancy in Shenzhen, China.	**Design:** study with data from the Congenital Syphilis Prevention Program. **Site:** SPPCS of the municipality of Shenzhen, including 90 prenatal clinics, of which 52 are public clinics and 38 are private clinics in the city. **Participants:** pregnant women identified with seropositivity for syphilis in their first prenatal consultation and had information about their treatment status. Pregnant women included in the analyses were limited to women whose babies had a definitive outcome of confirmation or exclusion of the diagnosis of congenital syphilis until October 2016.	VI
Nunes, JT, et al, 2017^(23)^	*Revista de Enfermagem - UFPE online*	Discuss nurses’ actions in prenatal care for pregnant women with syphilis and identify difficulties encountered by professionals in complying with treatment of pregnant women and partners.	**Design:** qualitative, descriptive-exploratory study. **Site:** *Unidade Mista de Felipe Camarão* (UMFC), Natal, Rio Grande do Norte, Brazil. **Participants:** four nurses who work in the care of pregnant women during prenatal care at the study site.	VI
Kidd S, et al, 2018^(24)^	Journal of the American Sexually Transmitted Diseases Association	Estimate the proportion of potential cases of congenital syphilis avoided with current prevention efforts and develop a classification framework to better describe why reported cases were not avoided.	**Design:** data from national reports of cases of female syphilis and congenital syphilis from the Brazilian National Notifiable Diseases Surveillance System. **Site:** United States. **Participants:** reported cases of syphilis in pregnant women and reported cases of congenital syphilis.	VI
Anugulruengkitt S, et al, 2020^(25)^	Pediatrics International - Official journal of de Japan Pediatric Society	Determine congenital syphilis rate and identify gaps in prevention.	**Design:** retrospective review of medical records. **Site:** tertiary care center, King Chulalongkorn Memorial Hospital, Bangkok, Thailand. **Participants:** pregnant women with positive serology for syphilis and their babies.	VI

**Chart 3:** Synthesis of articles included in the review according to the definition of adequate and inadequate treatment, prevalence/incidence, characteristics of participants and treatment, and factors associated with inadequate treatment

Authors/year	Results					
	Definition of appropriate treatment	Definition of inappropriate treatment	Prevalence or incidence	Characteristics/profile	Treatment	Factors associated with inadequate treatment
RotchforK, et al, 2000^(17)^	Complete treatment of syphilis: three doses of penicillin at weekly intervals; appropriate treatment of syphilis: at least two doses of penicillin at weekly intervals.	Inadequate treatment: one dose or less of penicillin received.	158 positive tests, with the prevalence of syphilis estimated at 9%.	The mean age was 25 years. The mean gestational age at the first antenatal visit was 24 weeks, and a history of previous perinatal death was reported by eight (7%) of 115 previously pregnant women.	30 (19%) received no treatment for syphilis, 20 (13%) received one dose of penicillin, 12 (7%) received two doses, and 96 (61%) received the recommended three doses. Thus, 50 (32%) women were considered inadequately treated. The average number of doses received was 2.1. The average delay of diagnosis until the first dose of penicillin was 20 days, for the second dose, it was 27 days, and among those who completed treatment, the mean time to completion was 34 days. Partner treatment: 78 (86%) reported receiving a contact card. Of these, 70 (77%) reported informing their partners about the need for treatment, but only 24 (26%) reported being sure that they had received it	Among inadequately treated pregnant women, the mean gestational age at the first prenatal visit was 27 weeks versus 23.6 for those who were adequately treated (p < 0.0001). The mean number of penicillin doses received was 0.4 versus 2.9 (p < 0.0001), respectively. the median delay to the first dose of penicillin was 31 days versus 18 (p < 0.0001), respectively. The median gestational age at the first dose of penicillin was 31 weeks versus 26 (p 0.0003), respectively. The number of perinatal deaths was 11 deaths versus 4 (p<0.0001).
Mullick S, 2005^(18)^	Three doses of penicillin	-	188 women were considered positive for syphilis, a prevalence rate of 1.03%.	The mean age of pregnancy at the first prenatal visit was 26 weeks, with 17% presenting at 30 weeks or later. Few women attended consultation before 20 weeks of gestation (10.7%).	Of 186 (2 missing), 64.8% of women received all three doses, 5.8% received two doses, 13.2% received one dose, and 15.9% of women received no treatment. The mean time elapsed from the test to receiving the first dose of treatment was 34 days. The majority (81%) were treated for the first time after 14 days and almost a fifth (18%) waited at least 2 months for the test to start treatment.	The number of treatment doses was significantly associated with gestational age at the first visit (p = 0.029). Women who presented later in prenatal care were less likely to receive all three doses.
Brito ESV, et al, 2009^(19)^	VDRL (Venereal Disease Research Laboratory), which should be performed in the first and third trimesters of pregnancy, and cure control for pregnant women and partners with a positive diagnosis.	-	A total of 234 cases of congenital syphilis were recorded.	The highest proportion of congenital syphilis occurs among women over 20 years of age, with less than 8 years of education, and 83.25% of them underwent prenatal care.	Both women (89.96%) and their partners (92.11%) were inadequately treated. Also, 36.9% of the group did not control the cure of VDRL positive pregnant women, and the majority (78.3%) had problems to carry out the treatment of partners of pregnant women.	According to nurses, poverty and ignorance are the main barriers they face to perform adequate treatment of pregnant women and their partners, including laboratory tests (54.3%). The first is the main cause that prevents access to health services. As for the second, fear and lack of knowledge about sexually transmitted diseases motivate users to refuse treatment for infections, especially by their partners.
Zhu L, et al, 2010^(20)^	Primary, secondary and early latent syphilis: benzathine penicillin G (4.8 million units) intramuscularly in two doses (9.6 million units total) weekly. Late latent syphilis: benzathine penicillin G (2.4 million units) intramuscularly in three doses (7.2 million units total) weekly.	Cases of maternal syphilis that did not complete a full course of treatment were considered treated incompletely	A total of 1,471 cases of maternal syphilis (298.7 per 100,000 live births) were identified. The maternal syphilis rate was 156.2 per 100,000 live births in Shanghai residents and 371.7 per 100,000 live births in the migrant population.	Among the identified cases of syphilis, the mean age was 27.2 years. The majority were unemployed (888)	392 had incomplete treatment.	They were associated with low compliance with treatment, lower maternal education, and in the incomplete treatment group, only 24% had completed high school or higher education versus 76% in the complete treatment group (p <0.05), lower paternal education, whereas, in the incomplete treatment group, 26% completed high school or higher versus 74% in the full treatment group (p < 0.05) and an abnormal reproductive history, which occurred in 35.9% of incomplete treatment cases versus 64.1% in the full treatment group (p <0.05).
Chávarro MAS, et al, 2017^(21)^	Three doses of benzathine penicillin one week apart and receiving the last dose at least one month before childbirth.	-	There were 54 cases of congenital syphilis recorded with an incidence rate of congenital syphilis of 13.4 cases per 1,000 live births in 2014. In the following year, 55 cases of congenital syphilis were recorded, with an incidence of 15 cases per 1,000 live births.	There were 106 cases of congenital syphilis. The mean maternal age in the group of congenital syphilis cases was 22 years, and 25 in the control group. 6% of mothers with syphilis did not complete elementary school, and in the control group, 2%.	Of the 106 cases identified, 66 (62.3%) were born to women diagnosed in the postpartum period, indicating failure to diagnose, and 40 (37.7%) from women diagnosed during prenatal care, but who received treatment inappropriately.	In the logistic regression model, the factors related to treatment failure were newborns of mothers with ≤ 5 prenatal visits were 2.85 times more likely to fail in treatment, compared to those with more than 5 visits (95% CI: 1.29-6.28). Mothers aged ≤ 18 years were 4.07 times more likely to fail treatment compared to those aged over 18 years (95% CI: 1.43-11.57).
Hong F, et al, 2017^(22)^	Intramuscular benzathine penicillin G, regardless of disease stage, for at least 1 course (2.4 million units once a week for 3 consecutive weeks). Penicillin-allergic pregnant women were treated with erythromycin 500 mg orally 4 times a day for 15 days. Azithromycin should be given 500 mg orally once a day for 10 days. Ceftriaxone sodium injection of 1 g daily for 10 days should be administered.	-	162 babies were diagnosed with congenital syphilis, a general incidence of 3.41%. Among children born to women seropositive for syphilis and treated appropriately before pregnancy, the incidence was 0.22%. There were 159 cases of congenital syphilis in 3,519 babies born to women seropositive for syphilis during pregnancy, an incidence of 4.52%.	-	-	The results of the multivariate analysis showed that women with complete or incomplete primary education were 1.5 times more likely not to treat syphilis compared to those with high school and higher education. (95% CI: 1.17-1.92). Local residents were 1.38 times more likely not to treat syphilis compared to those who were not local residents (95% CI: 1.00-1.88). Those who consulted antenatal clinics in less developed areas were 1.62 times more likely to not be treated for syphilis compared to those who consulted antenatal clinics in more developed areas (95% CI: 1.38-1.91). Those who had their first prenatal visit at the 28^th^ week of gestation or later were 21.47 times more likely not to treat syphilis compared to those who had consultation in less than 28 weeks (95% CI: 18.07-25.50). HIV-infected mothers were 4.01 times more likely not to treat syphilis compared to non-infected mothers (95% CI: 1.08-14.93), and women who treated syphilis before their current pregnancy were 1.65 times more likely not to treat syphilis compared to those who did not previously treat (95% CI: 1.36-2.02).
Nunes, JT, et al, 2017^(23)^	Benzathelin penicillin, completed 30 days before childbirth, with partner being treated concomitantly.	-	-	-	-	From the speeches, categories emerged: *Actions of nurses in monitoring pregnant women with syphilis; Aspects that hinder the effectiveness in gestational syphilis treatment; Syphilis: notifiable disease; Temporary lack of medication necessary for treatment; Absence of a protocol to ensure that nurses provide care to pregnant women with syphilis; Low compliance of partners and pregnant women with treatment, reporting it to be quite painful*.
Kidd S, et al, 2018^(24)^	Receiving the appropriate penicillin regimen for the maternal stage of syphilis started at least 30 days before childbirth.	Mothers without relevant documentation from any of services deemed appropriate were considered to have not received treatment.	There were 628 reported cases of congenital syphilis in the United States in 2016.	-	Of the 2,508 pregnant women with syphilis, 2,208 (88%) received prenatal care at least 30 days before childbirth, 2,242 (89.4%) were tested for syphilis at least 30 days before childbirth, 1,928 (76.9%) received an adequate treatment regimen and started at least 30 days before childbirth, 48 (7.6%) mothers of reported cases of congenital syphilis received an appropriate treatment regimen for their syphilis stage and started at least 30 days before childbirth, and 580 (92.4%) did not.	The most common reason for not receiving adequate treatment started at least 30 days before childbirth was the lack of tests at least 30 days before childbirth (n = 266; 45.9% of those not treated properly; 42.4% of all cases). Eighty-eight mothers (15.2% of those not properly treated; 14.0% of cases) were tested for syphilis at least 30 days before childbirth, tested positive, but did not receive treatment at least 30 days before childbirth.
Anugulruengkitt S, et al, 2020^(25)^	-	Untreated syphilis, undocumented therapy, use of antibiotics other than benzathine penicillin, insufficient dosing regimen, inadequate serologic response to treatment (<4-fold reduction in nontreponemal titers within 3 months), or therapy not taken within 1 month of childbirth.	The rate of congenital syphilis was 115 cases (95% CI 78-164) per 100,000 live births.	The median maternal age was 21 years, and 12 (17%) had HIV co-infection. The median gestational age at the time of diagnosis of syphilis was 23 weeks, with 25 (36%) diagnosed during the third trimester, followed by 22 (32%) and 11 (16%) during the second and first trimesters.	Of 69 pregnant women, there were 28 (41%) women with inadequate treatment.	The most common cause of lack of treatment was delay or absence of prenatal care, 13 (19%). Six (8%) were not treated or had incomplete treatment due to recent infection near childbirth. Five (7%) did not receive adequate treatment due to failure to provide and follow up on treatment. For example, no system to call back for treatment despite positive test results, or loss of follow-up during referral or after starting treatment. Four (6%) had inappropriate treatment in terms of dosage and regimen.

Based on the findings, it is noted that articles^(17-18,21-22,25)^ bring as factors associated with inadequate treatment of syphilis during pregnancy clinical variables related to pregnant women, such as syphilis treatment before the current pregnancy and HIV infection. Articles^(19-22)^ point out sociodemographic aspects, such as low education, income and maternal age, which sometimes imply ignorance about the disease and, consequently, inadequate treatment.

Additionally, other studies^(17,23-25)^ indicate the issues of dispensing the drug, prescription and follow-up of treatment, such as temporary lack of medication, failures in prenatal care, including delay or absence of it, delay in receiving the 1^st^ dose of penicillin, lack of tests or treatment performed less than 30 days before childbirth/miscarriage and inappropriate prescribing, in terms of dosage and regimen. Partners’ low treatment compliance, including the report of being painful, was pointed out in two articles^(19,23)^.

## DISCUSSION

This review showed factors associated with inadequate treatment of syphilis during pregnancy related to clinical variables, sociodemographic aspects and care failures.

Inadequate prenatal care was indicated as the main factor responsible for the high incidence of congenital syphilis in a study conducted in Belo Horizonte, Minas Gerais^(26)^. The same was found in national study developed in 2011 and 2012, who pointed out cases of congenital syphilis associated with lower education, later initiation of prenatal care, that is, fewer consultations and fewer serological tests. It was found that pregnant women without any prenatal consultation are the ones with the highest prevalence of syphilis during pregnancy^(27)^, corroborating the findings of eight of articles analyzed in this study.

In another study carried out at the Maternity Hospital of Malvinas, Argentina, it was pointed out that the risk of having some type of failure in the diagnosis of maternal syphilis was related to specific factors, such as low maternal education and insufficient number of prenatal exams. Moreover, pregnancy before the age of 18 and having less than 5 prenatal consultations are factors that affect failure of gestational syphilis treatment^(28)^.

Research identified the occurrence of syphilis in pregnancy associated with less than eight years of education, 7.4 times more likely in women who did not have prenatal care, inadequate or not performed treatment (53.7%) and 64.0% of the cases there was no treatment of their sexual partners^(4)^.

The epidemiological bulletin of HSD/MoH, brings that in 2020, 41.8% of women were diagnosed in the first trimester, 21.9% in the second trimester, and 30.1%, in the third. Still considering 2020, it was observed that more than half (56.4%) of pregnant women were between 20 and 29 years old when diagnosed with the disease, 23.3% between 15 and 19 years old and 17.3% aged between 30 and 39 years. Regarding education, most notifications (26.3%) were “ignored” the information, followed by 25.3% of pregnant women with elementary education^(29)^.

Maternal syphilis diagnosed late during pregnancy is considered a significant risk factor for congenital syphilis, as it implies late treatment or lack of treatment during pregnancy. It is reinforced that screening, diagnosis and timely treatment of syphilis are fundamental for the prevention of congenital syphilis and its adverse outcomes in pregnancy^(1)^.

Cases of congenital syphilis can be avoided by screening and treating pregnant women early, in addition to another assessments at the beginning of the third trimester to check for infections acquired during pregnancy^(30)^. Of the analyzed articles, only one^(24)^ indicated as a factor associated with inadequate treatment the lack of prenatal examinations less than 30 days before childbirth.

Regarding the maternal variables related to cases of congenital syphilis the maternal age of 20 to 25 years was evidenced, predominance of mothers with incomplete elementary education, at the height of menacme and residents of the urban area^(31-32)^. On the other hand, a study showed that almost all cases of syphilis in pregnant women had good compliance with prenatal care (96.6%), but despite this, almost 40% of pregnant women had the diagnosis during childbirth, and those who were diagnosed during prenatal care, less than half completed treatment less than 30 days before childbirth^(32)^.

Regarding partners’ treatment, only two articles^(19,23)^ addressed partners’ low compliance as a factor associated with inadequate treatment of syphilis in pregnant women, considering the definition of adequate treatment brought by the articles. In Brazil, despite the current Information Note 2 - SEI/2017 - DIAHV/HSD/MoH^(5)^ not considering the treatment of mothers’ sexual partners for the purpose of defining adequate treatment and a case of congenital syphilis, it is essential to consider that there is a risk of reinfection for pregnant women who are not treated concomitantly with partners.

A study conducted in Minas Gerais showed that only 34.3% of pregnant women and 19.8% of partners who underwent treatment for syphilis were considered adequately treated. It is emphasized that 176 (65.7%) of pregnant women had inadequate treatment or were not attended during prenatal care examinations. Patients with adequate treatment had lower rates of congenital syphilis when compared to those who were not treated^(33)^. Similar data were found in a study with secondary data in the city of Salvador, Bahia, where 49.3% of pregnant women did not undergo treatment properly, despite prenatal care and diagnosis during pregnancy. The article also states that 18.3% of pregnant women had incomplete elementary school and 39.6% of partners did not undergo treatment^(34)^.

Such findings converge with the articles analyzed in this review, which found, in most cases, high rates of inadequate treatment among pregnant women with syphilis, observing that structural problems still persist and limit the fight against congenital syphilis, which is a worrying fact that requires attention during prenatal care by health professionals in order to identify and minimize the factors that contribute to these results.

### Study limitations

Although the objective proposed by the study has been achieved, there are some limitations. The studies aggregated in this review refer to different cultural, social and economic realities and contexts that reflect in different actions and policies, as identified in the different definitions of inadequate treatment highlighted, in addition to the methodological variety, which made the comparative analysis of publications difficult.

This review showed that most studies focus on factors related to the prevalence of congenital syphilis in children of pregnant women who did not undergo treatment or did so improperly. However, it can be seen that a small number of articles directly, specifically and in depth on factors associated with inadequate treatment of pregnant women, the focus of our study, are mostly addressed in isolation and punctually. Therefore, it is necessary to expand research in this area in the various national and international scenarios that use homogeneous methodologies and representative samples in order to achieve a greater degree of evidence, thus filling in such gaps found in the preparation of this study.

### Contributions to nursing, health and public policies

Despite these limitations, by compiling and identifying the main factors related to inadequate treatment, the study presents advances for health and nursing, as it allows collaborating in the construction of improvement plans for prenatal care, allowing intervention in the face of the identification of such factors in practice with families during prenatal care, thus ensuring early treatment of syphilis both in pregnant women and in partners, and therefore preventing congenital syphilis. Finally, it is expected that the present study will foster further investigations, in order to fill the gaps found in the preparation of this study.

## FINAL CONSIDERATIONS

The findings pointed out as the main factors associated with inadequate treatment of syphilis during pregnancy, the clinical and sociodemographic aspects of pregnant women, as well as failures in drug dispensing, prescription and monitoring of treatment by the health system. Among these, coinfection (syphilis - HIV), history of treatment of the disease prior to current pregnancy, low education, maternal income and age, and low partner compliance with treatment stand out, in addition to temporary lack of medication, failures in prenatal care (absence or delay), including delay in receiving the 1^st^ dose of penicillin, lack of tests or treatment performed less than 30 days before childbirth/abortion and failures in prescriptions.

Thus, aiming to reduce the still high numbers of inadequate treatment of syphilis during pregnancy, with a consequent reduction in congenital syphilis and complications related to newborns, an integral and quality prenatal care is essential. To this end, policies to improve this assistance are necessary in order to guarantee, mainly, syphilis prevention, in addition to the early diagnosis and treatment of pregnant women and their partners.
